# Vision and Cognition: Findings from the Longitudinal Aging Study in India—Diagnostic Assessment of Dementia

**DOI:** 10.1093/geroni/igaa057.2281

**Published:** 2020-12-16

**Authors:** Joshua Ehrlich, Sandy Chien, Jinkook Lee

**Affiliations:** 1 University of Michigan, Ann Arbor, Michigan, United States; 2 University of southern california, Los Angeles, California, United States; 3 University of Southern California, Los Angeles, California, United States

## Abstract

Vision impairment (VI) is associated with cognitive decline and dementia, however little research has been conducted in India. Using data from LASI-DAD, linear models tested the association of VI (better-seeing eye <20/60) with cognitive domains including orientation, learning/memory, language, attention, and total cognition. Models were adjusted for age, sex, geographic, and socioeconomic characteristics. VI was significantly associated with lower orientation (β=-0.47, p<.01), learning/memory (β=-4.6, p<.01), attention (β=-1.6, p<.01), and total cognition (β=-8.4, p<.01), but not language (β=-0.14, p<.1) scores. The association of VI with cognitive measures did not vary by sex. For each measure, VI was equivalent to 5-13 years of cognitive aging. In summary, VI is associated with poorer performance in most cognitive domains among older Indian adults. Longitudinal data are needed to determine directionality and causality. Since >80% of VI in India is treatable, poor vision may represent a modifiable risk factor for cognitive decline and dementia.

